# Studying the Changes in Physical Functioning and Oxidative Stress-Related Molecules in People Living with HIV after Switching from Triple to Dual Therapy

**DOI:** 10.3390/antiox13050518

**Published:** 2024-04-26

**Authors:** Jessica Cusato, Anna Mulasso, Micol Ferrara, Alessandra Manca, Miriam Antonucci, Guido Accardo, Alice Palermiti, Gianluca Bianco, Francesco Chiara, Jacopo Mula, Maria Grazia Maddalone, Maria Cristina Tettoni, Simone Cuomo, Giulia Trevisan, Stefano Bonora, Giovanni Di Perri, Corrado Lupo, Alberto Rainoldi, Antonio D’Avolio

**Affiliations:** 1 Laboratory of Clinical Pharmacology and Pharmacogenetics, Department of Medical Sciences, University of Turin, Amedeo di Savoia Hospital, 10149 Turin, Italy; jessica.cusato@unito.it (J.C.); alice.palermiti@unito.it (A.P.); gianluca.bianco@edu.unito.it (G.B.); jacopo.mula@unito.it (J.M.); mariagrazia.maddalone@aslcittaditorino.it (M.G.M.);; 2 NeuroMuscolarFunction|Research Group, Department of Medical Sciences, University of Turin, 10128 Turin, Italy; anna.mulasso@unito.it (A.M.); simone.cuomo@unito.it (S.C.); corrado.lupo@unito.it (C.L.); alberto.rainoldi@unito.it (A.R.); 3 ASL Città di Torino, Amedeo di Savoia Hospital, 10149 Turin, Italy; micol.ferrara@aslcittaditorino.it (M.F.); miriam.antonucci@aslcittaditorino.it (M.A.); mariacristina.tettoni@aslcittaditorino.it (M.C.T.); 4 Unit of Infectious Diseases, Department of Medical Sciences, University of Turin, Amedeo di Savoia Hospital, 10149 Turin, Italy; guido.accardo@unito.it (G.A.); giulia.trevisan@unito.it (G.T.); stefano.bonora@unito.it (S.B.); giovanni.diperri@unito.it (G.D.P.); 5 Laboratory of Clinical Pharmacology S. Luigi A.O.U., Department of Clinical and Biological Sciences, University of Turin, Regione Gonzole, Orbassano, 10043 Turin, Italy; francesco.chiara@unito.it

**Keywords:** antioxidants, ROS, HAART, antiretroviral treatment, physical functioning

## Abstract

Background: Physical activity could increase the production of oxidative stress biomarkers, affecting the metabolism and excretion of antiretroviral drugs and, consequently, the clinical outcome. Nowadays, people living with HIV (PLWH) are mostly switching from triple to dual therapy, but no data are available in terms of physical functioning and oxidative stress. The aim of this study was to evaluate if some antioxidant biomarkers and physical functioning tests could be different according to triple or dual antiretroviral therapy. Methods: PLWH were evaluated at baseline (BL), while treated with three drugs, and six months after the switch to dual therapy. Physical functioning was quantified using validated tools. Mitochondrial and cytosol antioxidant molecules were evaluated through liquid chromatography. Results: Twenty-five patients were analyzed. A statistically significant difference between triple and dual therapy was found for mitochondrial glutathione, but not for physical tests. Evaluating differences between physically active and inactive individuals, the following statistically significant differences were suggested, considering triple therapy (mitochondrial n-formyl-methionine *p* = 0.022, triglycerides *p* = 0.023) and double therapy (mitochondrial glycine *p* = 0.035, cytosol glutamic acid *p* = 0.007, cytosol s-adenosylmethionine *p* = 0.021). Conclusions: For the first time, this study suggests possible differences in terms of antioxidant molecules and physical functioning in PLWH switching from triple to dual therapy.

## 1. Introduction

A significant improvement in life expectancy, with a decrease in acquired immune deficiency syndrome (AIDS)-related events, was observed with the introduction of antiretroviral therapy (ART) [1,2,3,4]. People living with human immunodeficiency virus (HIV), called PLWH, are basically treated with a combination of different antiretroviral drugs, possibly controlling their underlying disease [5].

Triple therapy is considered the gold standard for HIV viral replication control and for increasing life expectancy in PLWH [6]. These types of treatment generally include two nucleoside reverse transcriptase inhibitors (NRTIs, for example, lamivudine, emtricitabine, tenofovir, disoproxil fumarate, or alafenamide), in association with a protease inhibitor (PI, for example, ritonavir, fosamprenavir, cobicistat, or lopinavir), a non-nucleoside reverse transcriptase inhibitor (NNRTI, for example, rilpivirine or etravirine), or an integrase strand transfer inhibitor (INSTI, for example, dolutegravir, bictegravir, or elvitegravir) [7,8].

Possible side effects related to long-term combination ART can occur, requiring risk reduction strategies, but aging-related comorbidities, such as metabolic and renal disorders or cardiovascular diseases, are also typically observed [9,10]. In particular, in the literature, different studies highlighted an increasing incidence of cardiovascular risk in PLWH between 2015 and 2030, estimated with mathematical models [11]. Indeed, common adverse effects of antiretroviral drugs, such as NRTIs and PIs, are changes in body composition and lipid alterations in terms of whole body fat and peripheral, leg, and trunk fat, leading in some cases to lipodystrophy syndrome, which includes central lipohypertrophy, lipoatrophy of extremities, the face, and buttocks, and metabolic abnormalities [12]. For example, tenofovir disoproxil fumarate (TDF) is associated with renal and bone toxicity, and it has been mostly replaced with tenofovir alafenamide (TAF), associated with lower renal toxicity than TDF [13]. Despite this, TAF seems to show a possible relationship with weight gain and a less advantageous lipid profile [14,15,16,17], particularly in association with INSTIs [18,19,20].

In light of these data, reduction strategies such as dual therapy could lead to decreased toxicity and long-term adverse effects and, at the same time, allow for the maintenance of virological control in most cases [21,22], as reported in the literature [23].

Dual-therapy treatments were gradually introduced into guidelines globally, but despite this, *pros* and *cons* must be considered.

Dual therapy is generally composed of a NRTI-sparing ART or of a single NRTI associated with another antiretroviral [24]. Specifically, two of these regimens, dolutegravir (DTG) in association with lamivudine (3TC), both in naïve and suppressed patients, and DTG added to rilpivirine (RPV) in suppressed patients, have shown a great safety profile and efficacy [25,26,27].

The benefits of dual therapy are several: first of all, the lower number of tablets and the increased dosing intervals, which improve compliance, also represent a cost-effective choice. Moreover, decreased drug–drug interactions represent an additional *pro* of this line of treatment. It is known that tolerance and fewer long-term side effects are improved by prescribing a lower number of drugs. Finally, the exclusion of NRTIs in first-line treatments could spare these molecules for use in subsequent future regimens [28,29,30].

All these data are very important in this context, but, currently, there are no studies evaluating potential differences in physical functioning and oxidative stress in PLWH switching from triple to dual therapy.

On the other hand, the *cons* of dual regimens are possible increased virological failure and drug resistance. Additionally, viral escape is an important risk of suboptimal ART exposure [31,32,33]: triple therapies are the best therapeutic choice for adequate tissue penetrance and distribution of antiretroviral drugs, suppressing HIV replication. Regarding patients switching from triple to dual therapies, a longer follow-up to ensure viral escape is warranted. This precaution should be particularly carried out in subjects with a low CD4 count and/or a high baseline viral load; indeed, in these cases, virus replication in larger body compartments may occur, increasing suboptimal drug exposure and drug resistance [34]. Generally, the principal limitation of antiretroviral drugs is their inability to completely enter anatomic sanctuaries. For example, they did not obtain sufficient levels in lymphoid tissue, allowing replication and de novo infection of neighboring lymphocytes. Other examples of sanctuaries are the central nervous system and genitals. Incomplete viral suppression in these sanctuaries occurs for three-drug antiretroviral regimens but could be larger in dual-therapy regimens, such as maraviroc plus boosted darunavir administered once a day in the central nervous system. Some dual-therapy regimen limitations are that they are not generalizable, having proven substandard in patients with high HIV-1 RNA levels or low CD4 T-cell counts.

The safest and efficacious dual therapies, particularly as maintenance treatments, are reported in the article by Soriano et al. [35]. Currently, the combination of dolutegravir plus rilpivirine seems to be the best dual regimen, whereas longer follow-up and larger study populations are required before supporting this regimen. In contrast, dual therapy with maraviroc seems to be less effective. Although dual regimens with boosted protease inhibitors plus either lamivudine or raltegravir seem to be effective, they are associated with metabolic adverse events and the risk of drug interactions. The authors conclude that the newest dual regimens could save money, decrease toxicity, and spare drug options for the future.

Despite different studies focused on differences between triple and dual therapy in terms of clinical, virological, and immunological outcomes, interactions, and costs, no work has analyzed the difference in terms of oxidative stress. In fact, it is known that PLWH showed a reduced antioxidant state with a consequent high level of oxidative stress, particularly with low levels of reduced glutathione, called GSH, and an increase in its oxidized form, which is called GSSG. In 1996, a study showed a link between HIV-disease progression and GSH depletion [36,37]. Furthermore, a reduction in antioxidant enzymes (superoxide dismutase, SOD-1, and glutathione peroxidase, GPx) was suggested. Since PLWH have a reduced antioxidant state, they show an increase in HIV replication as a consequence of a reduction in immunological response [37]. Studies showed oxidative stress could have a potential impact on lipid and muscle metabolism, with a consequence on physical activity, also in PLWH [37,38].

Physical exercise has been used as a non-pharmacological therapy in order to improve anthropometrics, aerobic muscle, and physiological outcomes [39]. In 2020, Jankowski et al. measured the impact of exercise on HIV-positive and HIV-negative people, demonstrating that HIV-positive people were more likely to lose belly fat than gain muscle mass [38]. Some PLWH, who already present personal and environmental factors predisposing them to obesity, have a poor diet with high amounts of fat and sugar, chronic stress, a sedentary lifestyle, and negative lifestyle choices [40]. In this context, exercise could reduce chronic disease risk, inducing similar health benefits among PLWH. Indeed, most of the side effects from both the virus and ART (e.g., increased blood lipid profile, glucose tolerance, fatigue, chronic inflammation, anxiety, and depression) were improved with physical exercise. Regarding the immune system, it has been demonstrated that low-, moderate-, or high-intensity aerobic exercise does not negatively impact immune function or the progression of disease at any stage of the infection. Finally, only one study reported an increase in CD4+ cell count following a 16-week aerobic intervention [41]. It is important to highlight that ART could impact oxidative stress, but oxidative stress could also impact drug exposure. In fact, reactive oxygen species (ROS) and glutathione could influence the expression of genes encoding enzymes and transporters involved in antiretroviral drug absorption, metabolism, and excretion, and therefore the clinical outcome. Consequently, pharmacogenetics could influence transporter activity and thus antiretroviral drug exposure [42,43,44,45].

In addition, physical activity could lead to the production of ROS, which are known to affect some drug- and lipid-related transporters, including P-glycoprotein (P-gp or ABCB1) and ABCBA1 [46,47]. Specifically, it was demonstrated that ROS are able to downregulate P-gp expression [48]. This could impact anti-HIV drug exposure and, thus, on its efficacy or toxicity. ROS are oxygen intermediates with high reactive capacity towards various biological molecules [49]. They include hydroxyl radicals, superoxide anion, and hydrogen peroxide [50]. ROS are produced in various cellular processes and organelles: electron leakage from the mitochondrial electron transport chain, lipid degradation, or amino acid degradation [51]. Increased ROS levels are present in HIV-infected cell cultures [52,53]. PLWH has reduced antioxidant capacity, a decreased GSH/GSSG ratio in epithelial lung fluid, and a decreased GSH content in blood [49]. A study showed the number of CD4+ cells positively correlates with the total levels of ROS scavengers, such as GSH [54]. It is important to highlight that these changes are more pronounced in naive individuals compared to treated patients, since antiretroviral treatment restores the CD4+ number while increasing the redox status imbalance [55,56]. HIV-induced oxidative stress was shown to contribute to neurodegenerative complications, which are often observed in AIDS patients [49]. Only one study evaluated soluble inflammatory biomarker concentrations in PLWH, switching from triple to dual therapy (dolutegravir plus lamivudine), in 208 HIV-infected patients treated in a real-life setting. The authors found differences in mean log_10_ change from baseline to 48 weeks between the two therapies in terms of inflammation biomarkers such as interleukin-6, I-FABP, D-dimer, and CRP. The authors conclude that in a year, in this setting, I-FABP and CRP showed a favorable profile, switching to dolutegravir plus lamivudine compared to continuing a triple therapy.

Several studies evaluated differences in terms of efficacy, costs, side effects, and other factors in patients switching from triple to dual therapy [29,35,57,58]. For example, patients starting dolutegravir instead of triple therapy had a reduced risk of discontinuation for any reason [58].

Therefore, the aim of this study was to evaluate if some antioxidant biomarkers and physical functioning tests could be different according to the antiretroviral treatment in PLWH.

## 2. Materials and Methods

Treatment-naïve HIV-affected patients with an age between 30 and 50 years were recruited and evaluated before starting therapy (baseline, triple therapy) and after six months of therapy (dual therapy), possibly maintaining the same alimentary and physical habits. In this pilot study, patients were enrolled at the Unit of Infectious Diseases at Amedeo di Savoia Hospital (Turin, IT), from 2022 to 2023, and their hematochemical tests were reported. Patients administered potential interacting drugs were excluded; the same was true for co-infected patients.

Each participant signed an informed consent for storing blood samples for future analyses. This study was approved by the Ethics Committee (Study Prot No 17/2022, 16 March 2022, Comitato Etico Interaziendale Città della Salute e della Scienza, Turin, Italy). Physical activity was evaluated using the Global Physical Activity Questionnaire [59]. Physical function was measured using validated tools already administered to HIV patients (among these [60,61]), such as the tapping test for dexterity and the Sit-to-Stand test for leg strength. Also, anthropometric parameters (i.e., weight, height, BMI, waist circumference, and waist hip ratio) were monitored.

Antioxidant molecule levels were evaluated both in the cytosol and mitochondria through liquid chromatography.

All the considered variables were evaluated for normality through the Shapiro–Wilk test. Non-normal variables were resumed as median values and interquartile range (IQR); dichotomy variables as numbers and percentages.

Differences between linear and dichotomic variables (e.g., antioxidant molecules according to sedentary or not people) were evaluated through the Kruskal–Wallis and Mann–Whitney tests for unpaired samples and the Wilcoxon test for paired samples.

Tests were performed with IBM SPSS Statistics 28.0 for Windows (Chicago, IL, USA).

## 3. Results

### 3.1. Patient Characteristics

In this study, 25 patients were enrolled (9 sedentary and 16 non-sedentary): patient median age was 42.5 years (IQR 35.8–48) and median body mass index (BMI) was 23.3 kg/m^2^ (IQR 22.2–24.9).

The values of the hematochemical tests evaluated were reported in Table 1, while the administered drugs were reported in Table 2. No differences were highlighted between triple and dual therapies in terms of hematochemical values, with the exception OF vitamin D (*p* = 0.026).

Furthermore, patients always showed no detectable viral load, both in triple and dual therapy, maintaining the same lymphocyte count.

### 3.2. Cytoplasmic and Mitochondrial Factors of the Redox State Evaluation

**Figure 1:**
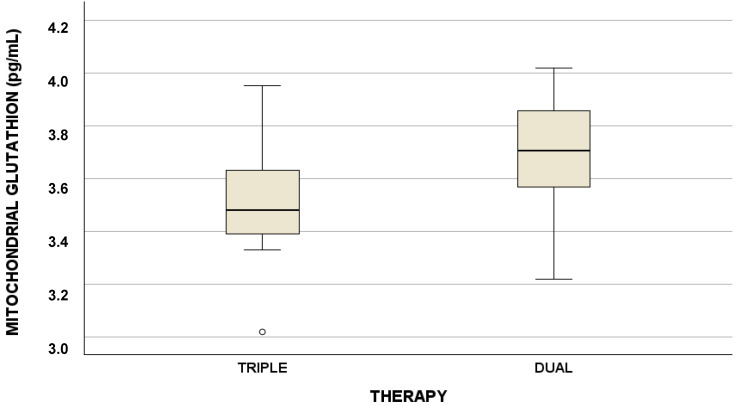
Role of triple and dual therapy in influencing mitochondrial glutathione concentrations (pg/mL).

The differences between the values of the cytoplasmic and mitochondrial factors of the redox state were evaluated between triple and dual therapy and reported in Table 3: a statistically significant difference for mitochondrial glutathione between triple and dual therapy was found. The respective influences of triple therapy and dual therapy on GSH levels ARE graphically reported in Figure 1.

Regarding GSH, the median was 3.5 pg/mL (IQR 3.4–3.6) for triple therapy and 3.7 pg/mL (IQR 3.6–3.9) for dual therapy.

### 3.3. Physical Functioning Evaluation and Differences between Physically Active and Inactive

The values of the physical functioning tests are reported in Table 4.

No statistically significant differences were suggested in terms of physical functioning tests between triple and dual therapy.

Since three subjects changed their physical activity, switching from triple to dual therapy, we performed these analyses on 22 people. We evaluated differences between physically active and non-physically active PLWH: in particular, statistically significant differences in hematochemical values and in cytoplasm and mitochondrial antioxidant components were suggested, considering triple therapy (mitochondrial n-formyl-methionine *p* = 0.022, triglycerides *p* = 0.023) and double therapy (mitochondrial glycine *p* = 0.035, cytosol glutamic acid *p* = 0.007, cytosol s-adenosyl methionine *p* = 0.021).

## 4. Discussion

Physical activity refers to movements produced by skeletal muscles; it requires energy use and includes movement during leisure time, for transport to get to and from places, or as part of a person’s work. Exercise is one form of physical activity, and it is a self-management strategy for improving health. Physical functioning refers to the capacity to perform different physical activities that are normal for people in good health. In this study, we did not find differences in physical functioning between people treated with triple therapy compared to PLWH treated with dual therapy [62].

Basically, PLWH are living longer, but some concomitant cardio-metabolic disorders are increasing due to viral processes, drugs, and physiological aging [63]. Compared to healthy subjects, PLWH often develop cardiovascular disease at a younger median age [64]. It is important to highlight that systematic physical exercise could decrease mortality from all causes by increasing cardiorespiratory and musculoskeletal fitness, balance, flexibility, or speed [62].

In addition, oxidative stress and inflammation are considered predictors of diseases associated with aging. In a study, markers of oxidative stress were analyzed in 213 PLWH on antiretroviral treatment to determine if they have an immunosenescent phenotype predisposing to the development of premature age-related pathologies. The concentrations of the oxidative stress biomarkers were not significantly different between untreated and treated patients. Furthermore, no significant associations were suggested between these biomarkers and CD4^+^ count, CD4^+^/CD8^+^ ratio, or HIV-1 RNA copies. Consequently, the authors conclude that highlighting high levels of oxidative stress-related molecules are independent of the virologic and immunologic status of PLWH. In conclusion, this study supports the hypothesis that residual viremia in cellular reservoirs of various tissues is related to the premature aging of the immune system and the predisposition to the premature development of aging pathology.

HIV-1 leads to oxidative stress by deregulating oxidative stress pathways and inducing mitochondrial dysfunction [49,52,53]. The enhancement of ROS production is mediated by Gp120, Tat, Nef, Vpr, and reverse transcriptase [65,66,67,68]. Particularly, both Gp120 and Tat suppress the expression of the glutathione synthesizing and metabolizing enzymes, such as glutathione synthase and glutathione reductase, leading to a decrease in the total glutathione content and an increase in the GSSG/GSH ratio [69]. Interestingly, Tat exhibits a stronger inhibitory effect on glutathione than Gp120 [49,69]. Mitochondrial dysfunction is a general mechanism of ROS production common to most viral infections [70,71]. NADPH oxidases and CYP2E1 serve as the major sources of ROS in infections with human hepatitis C, influenza, and respiratory syncytial viruses [49]. The overview of the field demonstrates that sources of ROS operational in HIV-1 infection follow similar trends; for example, a decrease in SOD3 activities was found in PLWH plasma [55].

In the works of Deresz et al. and Jankowski et al., the relationship between oxidative stress and HIV infection progression, along with the impact of physical exercise, was assessed, showing a link between HIV and oxidative stress [37,38].

Several studies evaluated differences in terms of efficacy, costs, side effects, and other factors in patients switching from triple to dual therapy [29,35,57,58], but no work investigated the dissimilarities in oxidative stress and physical functioning in these patients. Consequently, the aim of the present study was to evaluate the differences in antioxidant molecules and physical functioning tests in 25 patients switching from triple (baseline) to dual therapy (6 months), with 22 maintaining the same physical habits. Considering blood chemistry values, the therapy change has no effect on the antiviral treatment efficacy. This conclusion is supported by data on the lymphocyte population, in particular on T lymphocytes, whose total count in patients treated with dual therapy is comparable to that recorded at baseline. Furthermore, the HIV RNA demonstrates a significant reduction in viral load in both therapies, without differences between the two types of treatment.

As regards cytoplasmic and mitochondrial antioxidant factors, a difference between therapies was highlighted, indicating a possible influence of the therapeutic switch on oxidative stress. In particular, mitochondrial GSH is higher in dual therapy compared to triple therapy; this could be due to a reduced use of the detoxifying GSH molecule when using two drugs instead of three drugs. GSH is produced only in the cytosol, but it is also distributed in some intracellular components, including the nucleus, endoplasmic reticulum, and mitochondria [72]. The GSH compartmentalization suggests separate redox pools that are distinct from the cytoplasmic pool in terms of the balance of GSH/GSSG forms and their redox potential. Particularly in mitochondria, GSH is present mainly in its reduced form and represents a lower fraction of the total GSH pool (about 10–15%) [72]. It is important to highlight that mitochondrial function is closely linked to the maintenance of redox balance. In fact, mitochondria have a wide array of antioxidant and detoxifying enzymes, but they are the major source of ROS, most of which are produced from the mitochondrial respiratory chain. Conversely, the mitochondria are also targets for the ROS-damaging effect. Finally, toxic or pathologic conditions associated with an impairment of mitochondrial function can increase ROS release [72].

A sub-analysis on 22 subjects was performed, considering people maintaining the same physical habits and switching from triple to dual therapy. Differences between physically active and physically inactive individuals were highlighted, particularly in hematochemical values and cytoplasmic and mitochondrial antioxidant components. In triple therapy, mitochondrial n-formyl-methionine and triglycerides (sources of energy for the body) showed statistically significant differences, while in double therapy, mitochondrial glycine, cytosol glutamic acid, and cytosol s-adenosyl methionine.

Some of these molecules act on the energy production for the HIV replication cycle (cytosol glutamic acid), on increased oxidative stress and mitochondrial dysfunction (glycine mitochondrial), on the inflammatory activation of neutrophils (N-Formyl mitochondrial methionine), on the maintenance of cellular redox balance, and on the regulation of DNA methylation, a potential factor influenced by both HIV infection and antiretroviral therapy (s-adenosyl methionine mitochondrial).

Another consideration to be taken into account is that we highlighted higher vitamin D concentrations and reduced GSH in triple therapy, whereas reduced vitamin D levels and increased GSH in dual therapy: basically, higher vitamin D levels should be related to higher GSH levels [73]. The trend suggested in our work is opposite compared to the literature, probably due to other unknown potential impacting factors that are not analyzed in this context.

## 5. Conclusions

In conclusion, this study is the first to investigate the differences in terms of oxidative stress and physical functioning in PLWH switching from triple to dual therapy.

Particularly concerning cytoplasmic and mitochondrial antioxidant factors, a difference between therapies was highlighted, including mitochondrial higher GSH in dual therapy, indicating a possible influence of the therapeutic switch on oxidative stress.

This is a pilot analysis with the limit of a reduced sample size and a single cohort of analyzed patients; the study is following up on enrolling new patients and considering a larger time of analysis (e.g., one year or two years).

In addition, only men were enrolled in this study since no woman had the availability to participate. Consequently, further studies have to consider women in the study. Moreover, in the future, it would be interesting to evaluate the association between antiretroviral drug concentrations, particularly considering therapies currently administered, antioxidant molecules, and physical activity, as well as pharmacogenetics.

## Figures and Tables

**Table 1:** Levels of hematochemical parameters in triple and dual therapies.

	Triple Therapy		Double Therapy		
	MEDIAN	IQR	MEDIAN	IQR	*p*-Value
Weight	71.00	65.75–80.00	70.00	65.75–87.75	
White blood cells (wbc)	5.69	4.72–7.30	5.54	5.16–6.45	0.865
Red blood cells (rbc)	4.86	4.68–5.12	4.84	4.57–5.06	0.985
Hemoglobin (hgb)	153.50	144.30–158.0	151.50	140.3–157.50	0.690
Hematocrit (hct)	0.46	0.44–0.47	0.45	0.42–0.48	0.703
Platelets (plt)	265.00	215.00–301.00	239.00	192.5–289.3	0.478
Total lymphocytes %	76.56	69.95–83.10	75.40	67.4–80.7	0.413
Helper/inducer lymphocytes %	36.00	32.00–43.00	35.50	31.50–40.00	0.674
Suppressor/cytotoxic lymphocytes %	31.85	28.05–43.00	31.30	28.70–40.00	0.785
CD4/CD8	1.20	0.80–1.55	1.10	0.90–1.38	0.802
Glucose	84.00	78.00–86.0	80.0	73.50–87.80	0.634
Creatinine	1.00	0.89–1.09	1.01	0.90–1.14	0.521
Total cholesterol	189.50	153.0–203.80	179.00	164.00–203.00	0.869
High-density lipoproteins (hdl)	47.00	40.00–62.00	51.00	41.30–63.80	0.938
High-density lipoproteins (ldl)	118.00	91.00–130.00	107.50	100.80–137.50	0.938
Triglycerides	108.50	79.30–131.80	91	62.30–120.30	0.285
Aspartate amino transaminase (ast)	25.50	22.80–30.80	27.50	23.80–31.00	0.330
Alanine amino transaminase (alt)	27.50	20.50–33.80	28.00	20.00–34.00	0.553
Gamma glutamyl transpeptidase (ggt)	19.00	16.00–27.00	19.00	14.00–24.00	0.861
Alkaline phosphatase	62.00	52.00–82.00	63.50	56.80–73.80	0.938
Lactate dehydrogenase (ldh)	177.50	161.80–200.80	180.50	159.30–208.8	0.823
Creatinine kinase (ck)	134.50	89.80–192.50	159.50	96.00–220.8	0.409
Total bilirubin	0.49	0.38–0.67	0.46	0.39–0.59	0.726
Sodium	141.00	140.00–142.00	141.00	139.00–142.00	0.525
Potassium	4.20	4.07–4.53	4.30	4.20–4.50	0.399
Calcium	2.40	2.30–2.41	2.30	2.20–2.40	0.105
Phosphorus	3.05	2.80–3.50	3.10	2.80–3.43	0.930
Vitamin D	27.80	22.20–36.6	21.85	17.58–29.20	**0.026**
HIV RNA	Not detectable		Not detectable		**-**

**Table 2:** Drug regimens in enrolled individuals.

Drugs	Triple Therapy
DTG/ABV/3TC	1 (4%)
BIC/TAF/FTC	12 (48%)
DTG/TAF/FTC	1 (4%)
RPV/TAF/FTC	10 (40%)
DRV/c/TAF/FTC	1 (4%)
	**Dual Therapy**
DTG/3TC	20 (80%)
DTG/RPV	5 (20%)

**Table 3:** Levels of antioxidant molecules in triple and dual therapies.

	Triple Therapy		Double Therapy		
	MEDIAN	IQR	MEDIAN	IQR	*p*-Value
Mitochondrial cysteine	5.6	4.4–8.7	5.4	4.8–6.1	0.719
Mitochondrial glycine	25.03	17.8–35.6	26.1	18.5–33.6	0.719
Mitochondrial glutamic acid	11.2	9.4–13.1	11.1	9.9–13.3	0.379
Mitochondrial disolphorous glutathione	0.75	0.69–0.78	0.75	0.65–0.81	0.764
Mitochondrial glutathione	3.5	3.4–3.6	3.7	3.6–3.9	**0.003**
Mitochondrial homocysteine	1.2	1.1–1.6	1.2	1.1–1.5	0.826
Mitochondrial methionine	2.2	1.6–3.0	2.3	1.9–3.0	0.976
Mitochondrial n-acetyl cysteine	1.5	1.4–1.6	1.6	1.2–1.7	0.478
Mitochondrial n-formyl-methionine	4.5	4.1–4.9	4.5	4.1–5.0	0.569
Mitochondrial pyruvic acid	12.3	10.7–15.8	12.1	11.1–15.3	0.976
Mitochondrial serine	2.0	1.7–2.4	2.0	1.7–2.4	0.904
Mitochondrial taurine	2.0	1.1–2.2	1.9	0.9–2.2	0.881
Mitochondrial s-adenosyl methionine	0.11	0.08–0.13	0.12	0.10–0.13	0.207
Mitochondrial s-adenosyl homocysteine	0.0053	0.0039–0.0073	0.0055	0.0041–0.0066	0.849
Cytosol cysteine	3.9	3.6–4.8	4.2	3.8–4.5	0.285
Cytosol glycine	7.1	6.3–8.3	7.4	6.9–8.2	0.308
Cytosol glutamic acid	8.4	4.1–10.0	6.9	5.5–9.1	0.646
Cytosol disolphorous glutathione	0.62	0.52–0.66	0.61	0.54–0.65	0.795
Cytosol glutathione	35.0	25.3–56.1	33.6	25.8–57.6	0.834
Cytosol homocysteine	1.8	0.8–4.5	1.2	0.9–4.6	0.772
Cytosol methionine	2.9	2.1–3.2	3.1	2.6–3.7	0.267
Cytosol n-acetyl cysteine	3.2	2.9–3.6	3.2	2.7–3.5	0.810
Cytosol n-formyl-methionine	6.2	5.3–7.4	6.3	5.5–7.0	0.582
Cytosol pyruvic acid	15.6	12.6–17.4	14.5	12.9–17.0	0.976
Cytosol serine	3.0	2.1–3.2	2.7	1.9–3.3	0.490
Cytosol taurine	16.5	14.0–29.0	15.8	14.1–17.7	0.298
Cytosol s-adenosyl methionine	0.18	0.15–0.40	0.16	0.13–0.22	0.193
Cytosol s-adenosyl homocysteine	0.0280	0.0135–0.0570	0.0284	0.0171–0.0387	0.944

**Table 4:** Physical functioning evaluation in triple vs. dual antiretroviral therapies.

	Triple Therapy		Double Therapy		
Evaluated Factors	MEDIAN	IQR	MEDIAN	IQR	*p*-Value
Dominant tapping test	58	52–63	59	55–63	0.355
Non-dominant tapping test	54	51–58	54	48–59	0.778
Tapping test percentile	81	49–95	81	43–95	0.607
Dominant handgrip	44	34–46	46	43–49	0.101
Non-dominant handgrip	38	32–45	41	37–48	0.084
Handgrip percentile	31.5	10.3–46.3	37.5	30.0–62.5	0.121
Sit and reach	29	18–34	28	18–33	0.712
Sit and reach percentile	81	33–92	73	25–93	0.938
Sit to stand	6.14	5.38–6.90	5.65	4.42–6.06	0.103
Sit to stand percentile	69	55–80	82	63–90	0.145
Step test	104	88–119	96	80–114	0.277
Step test percentile	52	26–71	60	35–80	0.242

## Data Availability

The data are contained within the article.
